# Circulating Levels of Sclerostin Predict Glycemic Improvement after Sleeve Gastrectomy

**DOI:** 10.3390/nu13020623

**Published:** 2021-02-15

**Authors:** Federico Carbone, Elisa Nulli Migliola, Aldo Bonaventura, Alessandra Vecchié, Stefano De Vuono, Maria Anastasia Ricci, Gaetano Vaudo, Marcello Boni, Stefano Ministrini, Graziana Lupattelli, Fabrizio Montecucco

**Affiliations:** 1 First Clinic of Internal Medicine, Department of Internal Medicine, University of Genoa School of Medicine, 6 Viale Benedetto XV, 13132 Genoa, Italy; aldo.bonaventura@vcuhealth.org (A.B.); alessandra.Vecchie@vcuhealth.org (A.V.); 2 IRCCS Ospedale Policlinico San Martino Genoa—Italian Cardiovascular Network, 10 Largo Benzi, 16132 Genoa, Italy; fabrizio.montecucco@unige.it; 3 Internal Medicine, Angiology and Atherosclerosis, Department of Medicine and Surgery, University of Perugia, Piazzale Menghini, San’Andrea delle Fratte, 06132 Perugia, Italy; elisanullimigliola@yahoo.it (E.N.M.); insect3@tiscali.it (S.D.V.); m.anastasia.ricci@gmail.com (M.A.R.); gaetano.vaudo@unipg.it (G.V.); stefano.ministrini@studenti.unipg.it (S.M.); graziana.lupattelli@unipg.it (G.L.); 4 Department of Internal Medicine, ASST Sette Laghi, Viale Luigi Borri, 57, 21100 Varese, Italy; 5 Internal Medicine, “Santa Maria” University Hospital, Viale T. di Joannuccio, 05100 Terni, Italy; bonimarce@tiscali.it

**Keywords:** bariatric surgery, inflammation, insulin resistance, sclerostin, obesity

## Abstract

Among the different effects of bariatric surgery, here we focus on bone-derived inflammatory molecules, and in particular, sclerostin; an osteocyte product potentially associated with cardio-metabolic diseases. In 94 morbidly obese patients undergoing laparoscopic sleeve gastrectomy (SG), over-time changes in anthropometric and biochemical measures—including insulin resistance (IR) indexes—were correlated with serum sclerostin levels. Sclerostin was positively associated with anthropometric indexes of obesity, and inversely with IR, namely homeostatic model assessment for peripheral insulin sensitivity (HOMA2%S) (r = −0.218; *p* = 0.045). Sclerostin emerged as the only significant predictor of HOMA2-%S normalization, independently of demographic and anthropometric variables (OR 1.01 (95% CI 1.00–1.02); *p* = 0.024). We also identified two distinct patterns of serum sclerostin change: the higher/lower sclerostin levels at baseline, the greater their post-surgical reduction/increase (*p* < 0.001 for all subgroups). Among those two patterns, especially the post-surgery increase in serum sclerostin was associated with lean mass reduction, without any association with IR indexes. Although counterintuitive, this change was likely dependent on the post-surgical increase in bone turnover. In conclusion, baseline serum levels of sclerostin correlate with anthropometric measures of obesity and IR, and the ability to predict glycemic improvements after SG. Specifically, serum sclerostin was closely associated with peripheral insulin sensitivity (HOMA2-%S), thus supporting the role of skeletal muscle/bone interactions in metabolic diseases.

## 1. Introduction

Metabolic surgery in obese subjects is a validated approach for obtaining weight loss and health gain. The most relevant benefits seem to be related to its profound metabolic effects [1]. However, pathophysiological modifications following metabolic surgery are not fully clarified. Qualitative rather than quantitative changes in body fat composition are likely involved in metabolic improvements and these weight loss-independent mechanisms are increasingly attracting interest. They include favorable changes in gastrointestinal hormones, enterohepatic circulation and microbiota, but also a modified inflammatory status [2,3]. Recently, also bone metabolism has emerged as a potential player. Bone is now recognized as a fully active metabolic organ, with relevant implications in glucose metabolism [4]. Furthermore, bone density–and likely metabolism–are widely influenced by weight gain/loss and changes in muscle mass [5]. Bone-derived inflammatory molecules are well-recognized mediators of cardio-metabolic diseases [6] and some of them may also be synthetized and released by pro-inflammatory activated macrophages within dysfunctional visceral adipose tissue [7].

The present study was designed to investigate the role of sclerostin, an osteocyte product with suppressant activity on the osteo-anabolic Wnt/β-catenin pathway. Circulating levels of sclerostin were found to correlate with risk of fracture, homeostatic model assessment for insulin resistance (HOMA-IR), body mass index (BMI), and fat mass as well [8]. Here, we aimed at analyzing whether changes in serum levels of sclerostin occurred after metabolic surgery and potential correlations with metabolic parameters during one-year follow-up. Any potential predictive ability of sclerostin towards metabolic response to bariatric surgery has also been investigated, with particular regard to the glycemic profile.

## 2. Materials and Methods

### 2.1. Patient Enrollment

This is a sub-study of a previous published cohort enrolling morbidly obese patients undergoing laparoscopic sleeve gastrectomy (SG) [2]. From the original cohort of 110 morbid obese patients, serum samples were available for 94 patients, who were then included in this study. Since HOMA2-IR was not validated in individuals treated with exogenous insulin [9], 7 patients were excluded from the study cohort, which finally accounted for 87 patients. Inclusion and exclusion criteria are reported elsewhere [6]. The present study was approved by the local ethics board of Università degli Studi di Perugia (2014–2020), and all study subjects signed an informed consent form to voluntarily participate in this study. The study was registered at ClinicalTrials.gov with the following registration number: NCT03559842. The study was carried out in accordance with The Code of Ethics of the World Medical Association (Declaration of Helsinki) for experiments involving humans.

### 2.2. Anthropometric and Biochemical Assessment

Anthropometric parameters including weight, waist circumference, BMI, and hips circumference were completed by electric bioimpedance and ultrasonography measurement of visceral fat area (VFA). All measurements were performed before bariatric surgery (T0) and after 1 year of follow up (T1). Post-surgery changes were presented as delta (Δ) between T0 and T1 values. Electric bioimpedance (50 kHz, amplitude 50 mA, Body Composition Analyzer TBF-410GS; Tanita, Tokyo, Japan) was used to determine the fat mass as a percentage of body weight, whereas visceral fat (cm^2^) was measured by ultrasonography according to the Hirooka equation [10]. Blood samples were collected at T0 and T1 and drawn in the morning after 13-hour fasting state. A routine auto-analyzer was then used to assess lipids and glycemic profiles. Updated computer models for homeostasis model assessment 2 (HOMA2) (version 2.2.3 for Windows available from www.dtu.ox.ac.uk access date 1 April 2020) were also used to calculate IR (HOMA2-IR), insulin secretion (HOMA2-%β) and insulin sensitivity (HOMA2-%S). Visceral adiposity function (VAI) was calculated according to a previously validated equation [11]. Serum levels of sclerostin were measured by colorimetric enzyme-linked immunosorbent assay (ELISA) following the manufacturer’s instructions (R&D Systems, Minneapolis, MN). The lower limit of detection was 63 pg/ml whereas intra- and inter-assay coefficients of variation were <8%.

### 2.3. Study Endpoints

The primary outcome was to determine whether baseline sclerostin levels might predict any change in insulin sensitivity and overall glycemic profile during follow-up. More specifically, change in insulin sensitivity was defined by a shift across the cut-off level of 1.4 for HOMA2-IR and 100% for HOMA2-%S [12]. A change in glycemic profile was defined by the shift of glycemia from the range qualifying for type 2 diabetes to impaired fasting glycemia (IFG) or normoglycemia or by a shift from IFG to normoglycemia, according to recent expert opinion [13]. Normoglycemia was defined by a fasting glycaemia < 100 mg/dL or glycated hemoglobin (HbA1c) < 39 mmol/mol; IFG by fasting glucose ranging between 100–125 mg/dL (5.6–6.9 mmol/L) or HbA1c ranging between 39–47 mmol/mol. Diabetes was defined by a fasting glycemia ≥ 126 mg/dL (7.0 mmol/L) or HbA1c ≥48 mmol/mol in two determinations in absence of unequivocal hyperglycemia. As a secondary outcome, we tested the effectiveness of bariatric surgery in improving glycemic profile and insulin sensitivity in addition to report any correlation with post-surgery change in circulating levels of sclerostin. Two independent investigators adjudicated the study endpoints. Information was obtained during a check-up visit one year after surgery and further confirmed by checking patients’ medical records, targeting medical history relevant to the study endpoint.

### 2.4. Statistical Analysis

Analyses were performed using IBM SPSS Statistics for Windows, Version 23.0 (IBM CO., Armonk, NY). Categorical data are presented as relative and absolute frequencies. Continuous variables are expressed as the median and interquartile range (IQR), as the normality assumption was not demonstrated. Unpaired intergroup comparisons were drawn by Fisher’s exact test and Mann–Whitney *U*-test, whereas McNemar and Wilcoxon tests were used for paired data. Spearman’s rank test was then performed to investigate the correlation between sclerostin and other continuous variables. Linear regression analyses to confirm independent associations were also performed. Finally, univariate and multivariate (for age, sex, weight loss, Δ BMI, Δ waist circumference, Δ VFA, and Δ lean mass) logistic regression analysis was used to estimate the independence of the relationship between serum sclerostin levels and improvement of insulin sensitivity. Results were expressed as relative risk (RR) with 95% CI. For all statistical analyses a 2-sided *p*-value <0.05 was considered as being statistically significant.

## 3. Results

### 3.1. Metabolic Surgery Is Associated with an Increase in Circulating Levels of Sclerostin

Clinical and biochemical characteristics of the overall cohort at baseline (T0) and 1 year after SG (T1) were reported in Table S1. Whereas the prevalence of males was quite low (26.4%, *n* = 23), dyslipidemia was recognized in half of patients (51.7%, *n* = 45) and hypertension in 35.6% (*n* = 31). As expected, SG was associated with a huge improvement in anthropometric indexes in terms of weight, BMI, waist and hip circumference, and waist-to-hip/waist-to-height ratios (*p* < 0.001 for all). Electric bioimpedance analysis and ultrasound evaluation confirmed the loss of fat mass (both visceral and subcutaneous) along with a decrease in lean mass (*p* < 0.001 for all). Along with anthropometric indexes, SG significantly improved the metabolic profile, especially concerning glycemic control (Table S2). Finally, we reported a significant increase in circulating levels of sclerostin (from 14.5 at T0 to 118.9 pg/mL at T1 with a *p*-value <0.001).

### 3.2. Circulating Levels of Sclerostin Are Independently Correlated with Adipose Tissue Depots and Glycemic Profile at Baseline

**Figure 1:**
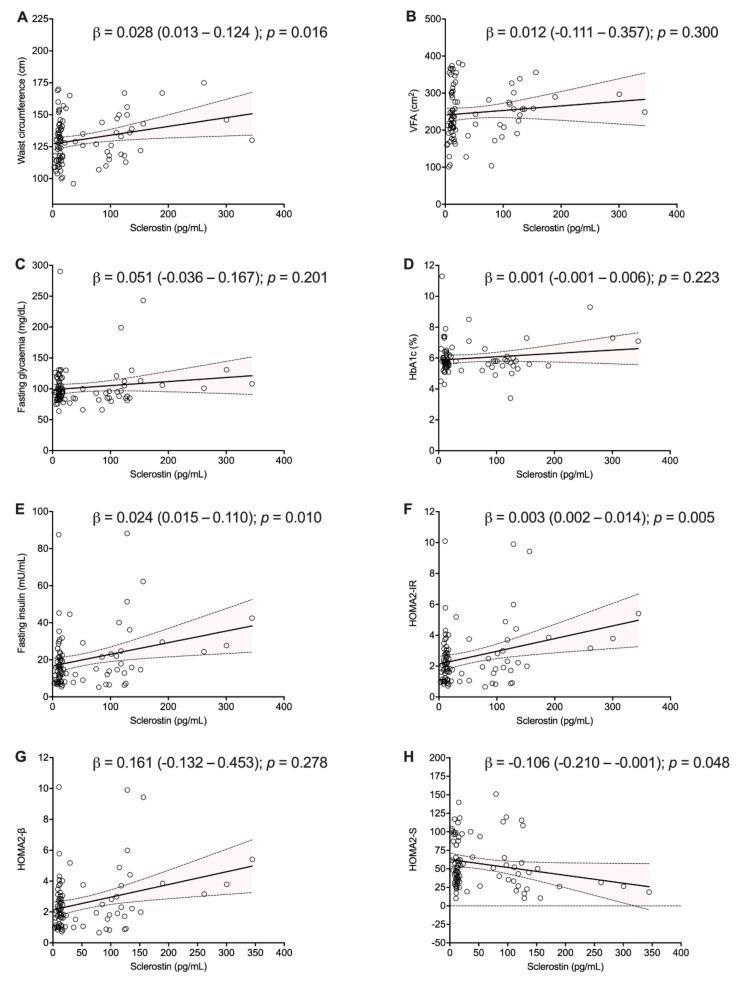
Serum sclerostin is independently associated with anthropometric index of fat mass and impaired insulin sensitivity. Linear regression analysis showing the association of sclerostin with waist circumference (**A**) and ultrasound-assessed visceral fat mass (VFA) (**B**). We also analyzed the potential associations with indexes of impaired insulin sensitivity: fasting glycemia (**C**), glycated hemoglobin (**D**), fasting insulin (**E**) and the homeostatic model assessment (HOMA)2 for insulin resistance (IR) (**F**), β cell function (β) (**G**) and peripheral insulin sensitivity (S) (**H**).

Sclerostin was found to generally correlate with fat mass (Table S3 and Figure 1). Significant direct correlations were observed for waist circumference (r = 0.238; *p* = 0.026), waist-to-hip/waist-to-height ratios (r = 0.315; *p* = 0.005 and r = 0.234; *p* = 0.029, respectively), while only a positive trend was observed for body weight and BMI. Additionally, electric bioimpedance analysis and ultrasound evaluation confirmed such relationship between sclerostin and fat mass (r = 0.218; *p* = 0.044), as well as both visceral and subcutaneous fat areas (r = 0.220; *p* = 0.045 and r = 0.228; *p* = 0.039, respectively). According to anthropometric measurements, significant correlations also emerged between sclerostin and IR, determined by insulin levels (r = 0.277; *p* = 0.010), HOMA2-IR (r = 0.300; *p* = 0.005) and HOMA2-%S (r = −0.218; *p* = 0.045). Linear regression analysis confirmed these associations, especially for waist circumference (β = 0.028; *p* = 0.016), HOMA2-IR (β = 0.003; *p* = 0.005) and HOMA2-S (β = −0.106; *p* = 0.048) (Figure 1).

### 3.3. Post-Surgical Variations in Serum Levels of Sclerostin Are Associated with Lean Mass Reduction and Insulin Resistance Improvement

**Figure 2:**
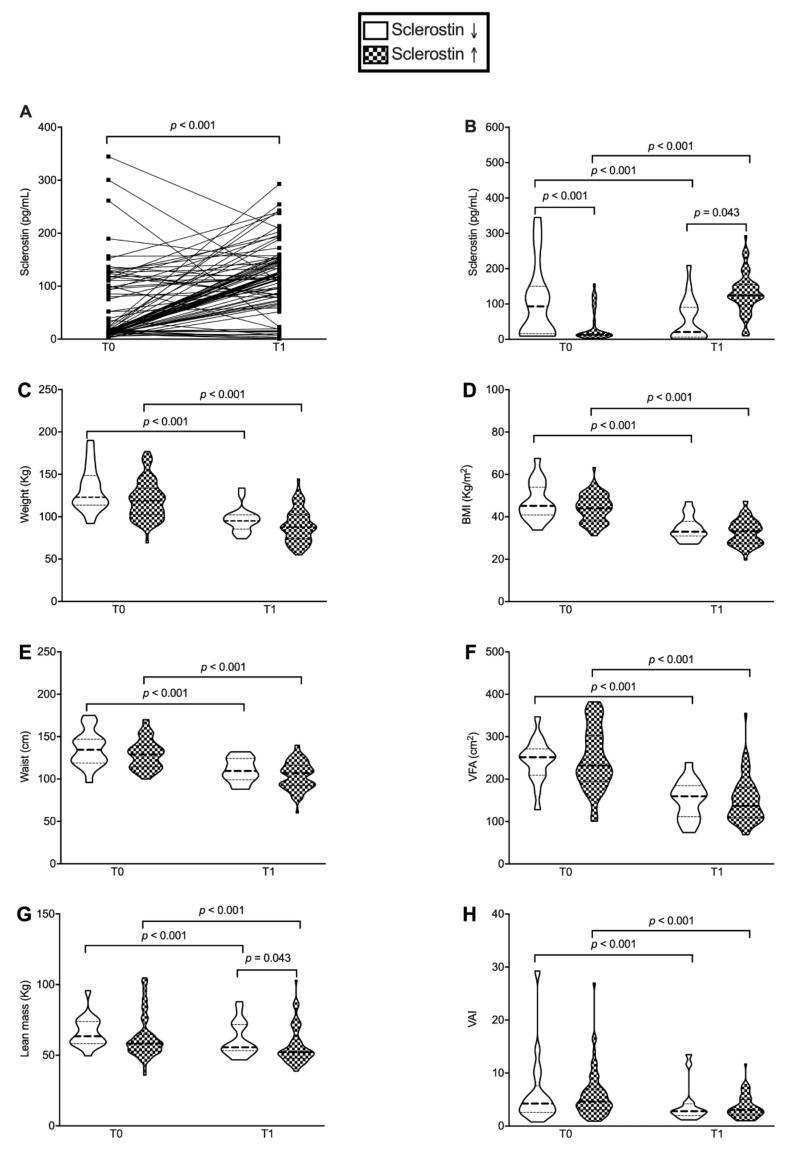
Different clinical response according to post-surgical change in serum sclerostin. These subgroups (**A**,**B**) did not show significant differences in anthropometric parameters (weight, body mass index (BMI), waist circumference, visceral fat area (VFA) and visceral adiposity index (VAI)), except for lean mass (**C**–**H**).

Δ sclerostin did not correlate with post-surgery modifications in anthropometric or biochemical variables (data not shown). However, we identified two distinct subgroups of patients in whom sclerostin decreased (*n* = 18) or increased (*n* = 69) (Figure 2A,B).

**Figure 3:**
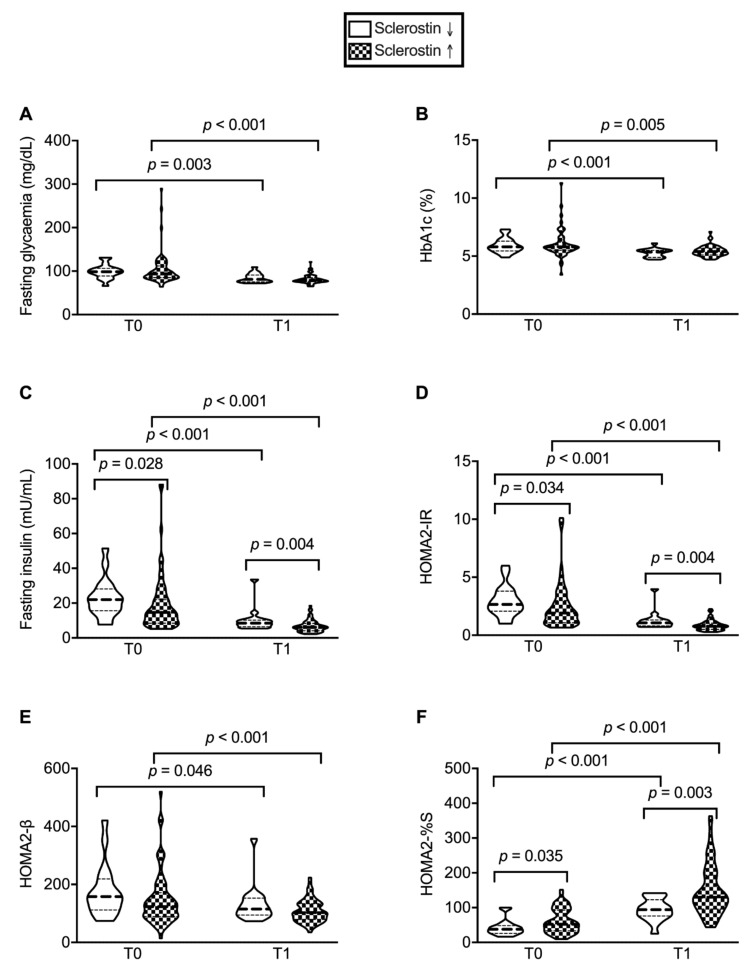
Different glycemic response according to post-surgical changes (decrease vs. increase) in sclerostin levels. Although all patients experienced an improvement in glycemic profile assessed by fasting glycemia (**A**) and glycated hemoglobin (HbA1c) (**B**), higher baseline sclerostin levels—but not post-surgical modification—are associated with higher insulin (**C**), homeostatic model assessment (HOMA)2 for insulin resistance (IR) (**D**), β cell function (β) (**E**) and peripheral insulin sensitivity (S) IR (**F**).

These groups significantly differed in their values at baseline (*p* = 0.001) and 1 year after SG (*p* < 0.001 for both subgroups). Accordingly, paired analyses confirmed a significant variation (*p* < 0.001 for both subgroups). Despite the different trend, post-surgical improvement of anthropometric parameters did not differ among these two subgroups, except for a slight—but statistically significant—reduction in lean mass in patients experiencing an increase in sclerostin levels (median values 52.2 vs. 55.7 Kg, *p* = 0.043) (Figure 2C−H). Similarly, all patients experienced an improvement in glycemic profile independently of sclerostin change, as assessed by fasting glycemia (*p* = 0.003 and *p* < 0.001, respectively) and HbA1c (*p* < 0.001 and *p* = 0.005, respectively) (Figure 3A,B).

As an additional finding, we confirmed that a worse glycemic profile was found in patients with higher baseline levels of sclerostin, insulin (*p* = 0.028), and HOMA2-IR (*p* = 0.003) and lower HOMA2-%S (*p* = 0.035). However, post-surgical reduction in sclerostin was not associated with any improvement of IR, but rather persistently higher levels of insulin (*p* = 0.004), HOMA2-IR (*p* = 0.0004) and lower levels of HOMA2-%S (*p* = 0.003) (Figure 2C–F).

### 3.4. Baseline Serum Levels of Sclerostin Are Negative Predictors of Worse Glycemic Recovery after SG.

**Figure 4:**
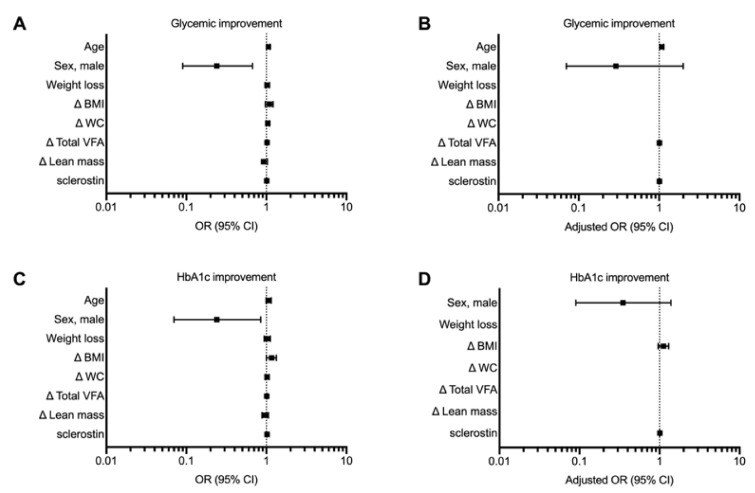

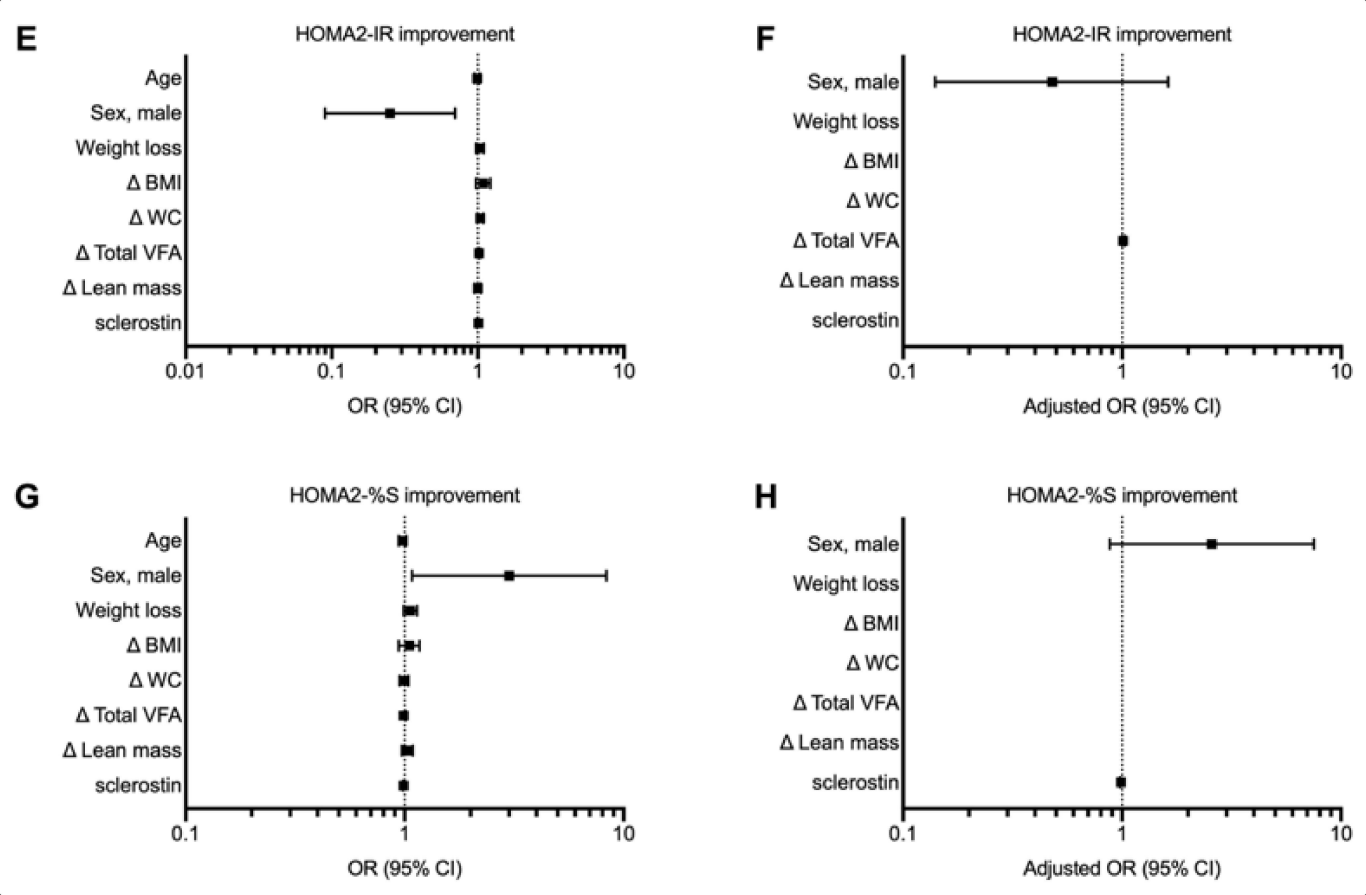
Forest plot illustrating potential determinants of glycemic improvement after sleeve gastrectomy. At univariate—but not adjusted—analyses, baseline values of sclerostin significantly predict glycemic profile improvement assessed by fasting glycemia (**A**,**B**) and glycated hemoglobin (HbA1c) (**C**,**D**), but not homeostatic model assessment (HOMA)2 for insulin resistance (IR) (**E**,**F**). Conversely, the predictive value of serum sclerostin toward an improvement in HOMA2-% for insulin sensitivity (S) was also confirmed at adjusted analysis (**G**,**H**). Other variables included in the model are body mass index (BMI), waist circumference (WC) visceral fat area (VFA) and lean mass.

As expected, SG was associated with an improvement in glycemic profile, with a general normalization of fasting glycemia and only a few cases of IFG (Figure S1A,B). Accordingly, the prevalence of normal HOMA2-IR and HOMA2-%S increased significantly (Figure 1C,D). When logistic regression analysis was performed, baseline values of sclerostin emerged as predictors of glycemic profile improvement, assessed by changes in fasting glycemia (OR 1.01 (1.00–1.02); *p* = 0.021) and HbA1c (OR 1.02 (1.01–1.02); *p* = 0.011). However, the predictive value of sclerostin decreased after adjustments for confounding factors (Figure 4A,C, and G for the unadjusted model and B, D, F and H for the adjusted model; Table S4).

Whereas no independent association was demonstrated with improvement of HOMA2-IR, sclerostin showed a significant predictive value for HOMA2-%S at regression analysis and also after adjustment (adjusted OR 1.01 (1.01–1.02); *p* = 0.024) (Figure 4E–H and Table S4).

## 4. Discussion

Partially confirming previous larger studies, here we attempted at highlight some unclear aspects of metabolic surgery. Firstly, we have observed a significant association between serum sclerostin levels and anthropometric indexes of obesity.

Humans exhibit a remarkable variability in body fat distribution and the susceptibility to store fat either subcutaneously or viscerally is only partially determined by genetics. The shift of visceral adipose tissue towards a dysfunctional phenotype–also referred to as “adiposopathy”–has been largely ascribed to a perturbation in the crosstalk with various organ systems, where ectopic fat deposition may occur (e.g. liver, pancreas, heart, and skeletal muscle) [14]. Since bone homeostasis is tightly linked to nutritional status and whole-body metabolisms, it is conceivable that bone related mediators may have a role in determining obesity phenotype and potentially unrecognized effects on metabolic response to bariatric surgery. In this regard, a general agreement exists about the role of chronic low-grade inflammation [15], that influences metabolic response to bariatric surgery [2] and even long-term diabetes remission [16,17]. Whether bone homeostasis may contribute to the remarkable variability in body fat distribution and storing is less evident.

Even more interesting, we observed a close relationship with HOMA2-IR, further characterized by an inverse correlation with insulin sensitivity (as assessed by HOMA2-S) but not HOMA2-β cell function. These results are confirmative of previously reported studies with pre-diabetes and overt diabetes settings [8,18], especially concerning the selective correlation with peripheral insulin sensitivity [19]. Later studies on *sost* knockout mice and subjects with prediabetes have confirmed the beneficial role of Wnt signaling inhibition in adipocytes, with protective effects from diet-induced obesity and IR [20,21]. Conversely, sclerostin deletion would not affect insulin sensitivity in liver, whereas the effect on skeletal muscle would be independent on Wnt signaling. [20,22]. However, this remains a controversial point as an interplay between Wnt and insulin signaling pathways in skeletal muscle has been reported elsewhere in humans [23]. Anyway, given the fact that adipocytes do not express *sost* gene [20], sclerostin should be considered as paracrine/endocrine effector of bone metabolism with potential role in glucose metabolism. In line with that, baseline serum levels of sclerostin have been here described as independent predictor of HOMA2-%S normalization after 1 year of follow-up. Such specificity for peripheral insulin sensitivity may be considered the major strength of the study but confirmatory studies are needed. 

Our results also provided relevant information about the effects of metabolic surgery on adipose tissue dysfunction. Quantitative and qualitative changes in visceral and ectopic fat composition has been documented in magnetic resonance studies [9,24,25,26] and even molecular signatures of metabolic surgery have been recently described in the adipose tissue [27]. There would be then the opportunity for new adiposity-related biomarkers to emerge and address some of the current shortcomings in metabolic surgery. Here, we observed a general increase of circulating sclerostin levels during the first year after bariatric surgery [28]. Nevertheless, we were able to identify two distinct sub-groups of patients with opposite trend. A sub-group of patients showed decreased sclerostin levels after surgery, characterized by higher sclerostin levels at baseline. Conversely, subjects with a lower baseline level of sclerostin, experienced a larger increase. Of interest, post-surgical increase in sclerostin levels was associated with lean mass reduction. This is not surprising as sclerostin is intimately related to muscular tension and reduced by mechanical stimulation [29,30,31]. However, our finding points out–and potentially extends–the concept of skeletal muscle/bone interaction in cardio-metabolic disease. The sarcopenic obesity phenotype may be considered the paradigm of this concept [32], being characterized by a concomitant overriding of abnormal muscle loss–usually age-dependent–and fat accumulation [33]. Whether the bone is implicated is still controversial, but someone argued that some osteosarcopenic obesity phenotype would exist [34,35].

As additional finding, patients experiencing post-surgical reduction of sclerostin levels did not show any improvement of IR but persistently high HOMA2-IR and low HOMA2-%S. This observation seems counterintuitive and deserves further investigations. Indeed, metabolic surgery usually determines an immediate increase of bone turnover and the consequent decline in bone mineral density is associated with the increase of specific biomarkers including sclerostin [28,36]. However, this is a multi-factorial process that seems only partially dependent of weight loss [37].

Some limitations have to be considered in interpreting our results. Due to the limited sample size and the sub-study design, we could not perform an adequate study power calculation and therefore our results should be considered as preliminary. In addition, the sub-study design did not allow the collection of data on important variables such pre-/post-menopausal status in females, post-surgical changes in bone mineral density and other related biomarkers and patient categorization according to the sarcopenic and osteosarcopenic obesity phenotypes. The cutoff point definition for HOMA2 indexes represents another limitation as they have not yet been clearly standardized. Fourthly, we acknowledge that glycemic normalization at one year after bariatric surgery is not a long enough time to define a remission from diabetes. Finally, our results are limited to clinical observation without providing any causal relationship between serum sclerostin levels and pre-/post-surgical glycemic profile.

## 5. Conclusions

In conclusion, baseline serum levels of sclerostin correlate with anthropometric measures of obesity and insulin resistance and can predict glycemic improvements after SG. More specifically, a tight association with baseline peripheral insulin sensitivity and its post-surgical normalization was observed. Although our results need validation in larger studies and mechanistic explanation, assessing serum sclerostin might have potential clinical implications.

## Data Availability

Data available on request due to privacy restrictions.

## Supplementary Materials

The following are available online at https://www.mdpi.com/2072-6643/13/2/623/s1, Table S1: Clinical and biochemical characteristics of the overall cohort at baseline (T0) and their post-surgical variations (T1) (*n* = 87); Table S2. Glycemic profile and circulating sclerostin (SOST) in the overall cohort at baseline (T0) and their post-surgical variations (T1) (*n* = 87); Table S3: Correlations of sclerostin with clinical, biochemical and inflammatory parameters; Table S4: Logistic regression linking baseline sclerostin with post-surgical change in glycemic profile; Figure S1: Sleeve gastrectomy significantly improves glycemic profile.
