# Supplementary material for: Circulating Levels of Sclerostin Predict Glycemic Improvement after Sleeve Gastrectomy
Source: Nutrients. 2021 Feb 15;13(2):623. doi: 10.3390/nu13020623 (PMC7918938; doi:10.3390/nu13020623)
Supplement: Supplementary file 1 [file nutrients-13-00623-s001.pdf]

Supplementary Material

Table S1. Clinical and biochemical characteristics of the overall cohort at baseline (T0) and their post-surgical variations (T1) (n=87).

**Demographic**	**T0**	**T1**	**Δ T0-T1**	***p*-value**
Age, yr. [IQR]	44 [35 – 53]	–	–	–
Sex, male (%)	23 (26.4)	–	–	–
Hypertension, n. (%)	31 (35.6)	–	–	–
Dyslipidemia, n. (%)	45 (51.7)	–	–	–
T2DM, no (%)	8 (9.2)	–	–	–
Morbidly obese, n. (%)	64 (73.6)	–	–	–
Active smokers, n (%)	23 (26.4)	–	–	–
**Clinical Data**				
Systolic BP, mmHg [IQR]	138 [130 – 150]	126 [118 – 130]	15 [3 – 22]	**<0.001**
Diastolic BP, mmHg [IQR]	88 [80 – 90]	80 [70 – 82]	10 [0 – 20]	**<0.001**
Weight, Kg [IQR]	120 [105 – 138]	88.1 [75.9 – 102.9]	29.4 [24.5 – 37.5]	**<0.001**
BMI, Kg/m^2 [IQR]	45.0 [38.8 – 50.0]	33.2 [27.9 – 36.9]	11.0 [8.9 – 13.7]	**<0.001**
Waist circumference, cm [IQR]	129 [116 – 143]	107 [94 – 118]	24 [19 – 32]	**<0.001**
Hip circumference, cm [IQR]	139 [130 – 148]	114 [104 – 125]	22 [17 – 30]	**<0.001**
Waist-to-hip ratio, [IQR]	0.94 [0.88 – 0.98]	0.10 [0.85 – 0.97]	0.03 [-0.01 – 0.06]	**<0.001**
Waist to height ratio, [IQR]	0.79 [0.72 – 0.86]	0.64 [0.58 – 0.70]	0.15 [0.11 – 0.19]	**<0.001**
**Electric bioimpedance analysis**				
Fat mass, Kg [IQR]	57.5 [45.5 – 67.3]	32.7 [23.8 – 41.4]	23.1 [19.3 – 41.4]	**<0.001**
Lean mass, Kg [IQR]	59.2 [53.8 – 68.2]	53.4 [49.5 – 63.0]	5.8 [3.4 – 8.3]	**<0.001**
**Ultrasound assessment**				
Total visceral fat area, cm^2 (IQR)	240 [205 – 297]	150 [109 – 181]	96 [60 – 134]	**<0.001**
Subcutaneous fat area, cm^2 (IQR)	30 [23 – 38]	18 [14 – 25]	11 [5 – 25]	**<0.001**
Visceral to subcutaneous fat ratio, (IQR)	8.1 [6.3 – 11.3]	7.5 [5.9 – 9.4]	0.4 [-1.8 – 2.6]	0.262
**Biochemical parameters**				
GFR, mL/min (IQR)	116 [105 – 133]	116 [95 – 132]	3 [-12 – 14]	0.375
Serum total-c, mg/dL [IQR]	194 [173 – 219]	190 [167 – 213]	5 [-16 – 21]	0.369
Serum LDL-c, mg/dL [IQR]	108 [96 – 130]	113 [67 – 132]	-1 [-20 – 12]	0.499

Serum HDL-c, mg/dL [IQR]	49 [43 – 59]	56 [47 – 64]	-6 [-13 – 1]	**<0.001**
Serum TAG, mg/dL [IQR]	124 [88 – 188]	95 [75 – 120]	30 [0 – 87]	**<0.001**
VAI, [IQR]	4.4 [2.7 – 6.9]	2.8 [1.9 – 4.3]	1.4 0.3 – 3.3]	**<0.001**

Data are expressed as median [IQR] or number n (%). *p*-value refers to the comparison between T0 and T1. Statistically significant *p*-values are in bold.

T2DM: type 2 diabetes mellitus; BP: blood pressure, BMI: body mass index; GFR: glomerular filtration rate; total-c: total cholesterol; LDL-c: low-density lipoprotein cholesterol; HDL-c: high-density lipoprotein cholesterol; TAG: triglycerides, VAI: visceral adiposity index.

Table S2. Glycemic profile and circulating sclerostin in the overall cohort at baseline (T0) and their post-surgical variations (T1) (n=87).

	T0	T1	Δ	*p*-value
Fasting glycaemia, mg/dL [IQR]	95 (85 – 109)	79 (76 – 86)	16 (6 – 28)	**<0.001**
HbA1c, mmol/mol [IQR]	40 (37 – 44)	36 (32 – 38)	5 (4 – 8)	**<0.001**
Insulin, mU/mL [IQR]	16 (9 – 24)	6 (5 – 9)	9 (4 – 16)	**<0.001**
HOMA2-IR, [IQR]	2.1 (1.2 – 3.2)	0.8 (0.6 – 1.1)	1.2 (0.5 – 2.1)	**<0.001**
HOMA2-β, [IQR]	133.1 (93.7 – 176.9)	103.3 (83.4 – 134.2)	27.7 (3.3 – 60.0)	**<0.001**
HOMA2-S, % [IQR]	47.9 (31.8 – 84.8)	122.5 (89.3 – 169.7)	-68.2 (-100.5 – -42.7)	**<0.001**
Sclerostin, pg/mL [IQR]	14.5 [10.9 – 94.4]	118.9 [78.5 – 150.6]	-77.9 [-115.7 – -2.9]	**<0.001**

Data are expressed as median [IQR]. Statistically significant *p*-values are in bold.
HbA1c: glycosylated hemoglobin; HOMA: homeostatic model assessment for insulin resistance (IR), β cell function (β) and peripheral insulin sensitivity (S).

Table S3. Correlations of sclerostin with clinical, biochemical and inflammatory parameters.

	Sclerostin	
	r	*p*-value
**Clinical Data**		
Age	-0.026	0.808
Systolic BP*	-0.009	0.939
Diastolic BP	0.181	0.110
Weight	0.208	0.053
BMI	0.204	0.058
Waist circumference	0.238	**0.026**
Hip circumference	0.106	0.333
Waist-to-hip ratio	0.315	**0.005**
Waist-to-height ratio	0.234	**0.029**
**Electric bioimpedance analysis**		
Fat mass	0.218	**0.044**
Lean mass	0.209	0.053
**Ultrasound assessment**		
Total visceral fat area	0.220	**0.045**
Subcutaneous fat area	0.228	**0.039**
Visceral to subcutaneous fat ratio	-0.012	0.917
**Biochemical parameters**		
GFR, mL/min	0.030	0.787
Serum total-c	0.008	0.943
Serum LDL-c	0.045	0.682
Serum HDL-c	-0.076	0.488
Serum TAG	-0.038	0.729
VAI	-0.129	0.237
**Glycemic profile**		
Fasting glycaemia	0.077	0.478
HbA1c	0.134	0.250
Fasting insulin	0.277	**0.010**
HOMA2-IR	0.300	**0.005**
HOMA2-β	0.019	0.278
HOMA2-S	-0.218	**0.045**

p-value refers to the correlations performed by Spearman's rank correlation coefficient. Statistically significant *p*-values are in bold.

BP: blood pressure; BMI: body mass index; GFR: glomerular filtration rate; total-c: total cholesterol; LDL-c: low-density lipoprotein cholesterol; HDL-c: high-density lipoprotein cholesterol; TAG: triglycerides; VAI: visceral adiposity index; HbA1c: glycosylated hemoglobin; HOMA: homeostatic model assessment for insulin resistance (IR), β cell function (β) and peripheral insulin sensitivity (S).

Table S4. Logistic regression linking baseline sclerostin with post-surgical change in glycemic profile.

	Univariate		Adjusted	
	**OR (95% CI)**	***p*-value**	**OR (95% CI)**	***p*-value**
**Fasting glycemia improvement**				
Age	1.06 (1.01 – 1.11)	**0.016**	1.08 (1.02 – 1.14)	**0.008**
Sex, male	0.24 (0.09 – 0.67)	**0.006**	0.29 (0.07 – 1.14)	0.077
Weight loss	1.02 (0.96 – 1.09)	0.501	–	–
Δ BMI	1.09 (0.97 – 1.21)	0.140	–	–
Δ waist circumference	1.04 (0.99 – 1. 09)	0.109	–	–
Δ Total visceral fat area	1.02 (1.01 – 1.03)	**0.004**	1.01 (0.99 – 1.02)	0.072
Δ Lean mass	0.92 (0.92 – 1.03)	0.377	–	–
Sclerostin	1.01 (1.00 – 1.02)	**0.021**	1.01 (0.99 – 1.02)	0.072
**Improved HbA1c**				
Age	1.07 (0.99 – 1.15)	0.052	–	–
Sex, male	0.24 (0.07 – 0.85)	**0.027**	0.35 (0.09 – 1.39)	0.350
Weight loss	1.03 (0.94 – 1.12)	0.516	–	–
Δ BMI	1.16 (1.00 – 1.33)	**0.048**	1.12 (0.96 – 1.30)	0.157
Δ waist circumference	1.02 (0.96 – 1. 08)	0.599	–	–
Δ Total visceral fat area	1.01 (1.00 – 1.03)	0.051	–	–
Δ Lean mass	0.97 (0.88 – 1.06)	0.966	–	–
Sclerostin	1.02 (1.01 – 1.02)	**0.011**	1.01 (1.00 – 1.02)	**0.037**
**Improved HOMA2-IR**				
Age	1.02 (0.98 – 1.06)	0.319	–	–
Sex, male	0.33 (0.12 – 0.93)	**0.035**	–	–
Weight loss	0.94 (0.88 – 1.00)	0.060	–	–
Δ BMI	0.95 (0.86 – 1.06)	0.381	–	–
Δ waist circumference	1.00 (0.96 – 1. 05)	0.903	–	–
Δ Total visceral fat area	1.00 (0.99 – 1.01)	0.635	–	–
Δ Lean mass	0.97 (0.92 – 1.03)	0.306	–	–
Sclerostin	1.01 (0.99 – 1.01)	0.522	–	–
**Improved HOMA2-%S**				
Age	0.98 (0.94 – 1.02)	0.319	–	–

Sex, male	3.00 (1.08 – 8.34)	**0.035**	2.57 (0.88 – 7.52)	0.084
Weight loss	1.06 (0.99 – 1.14)	0.060	–	–
Δ BMI	1.05 (0.94 – 1.17)	0.381	–	–
Δ waist circumference	0.99 (0.95 – 1.04)	0.903	–	–
Δ Total visceral fat area	0.99 (0.99 – 1.01)	0.635	–	–
Δ Lean mass	1.03 (0.97 – 1.09)	0.306	–	–
Sclerostin	1.01 (1.00 – 1.02)	**0.013**	1.01 (1.00 – 1.02)	**0.024**

Statistically significant *p*-values are in bold.
OR: odds ratio; CI: confidence interval; BMI: body mass index; HOMA: homeostatic model assessment for insulin resistance (IR) and peripheral insulin sensitivity (S).

Supplementary Figure 1

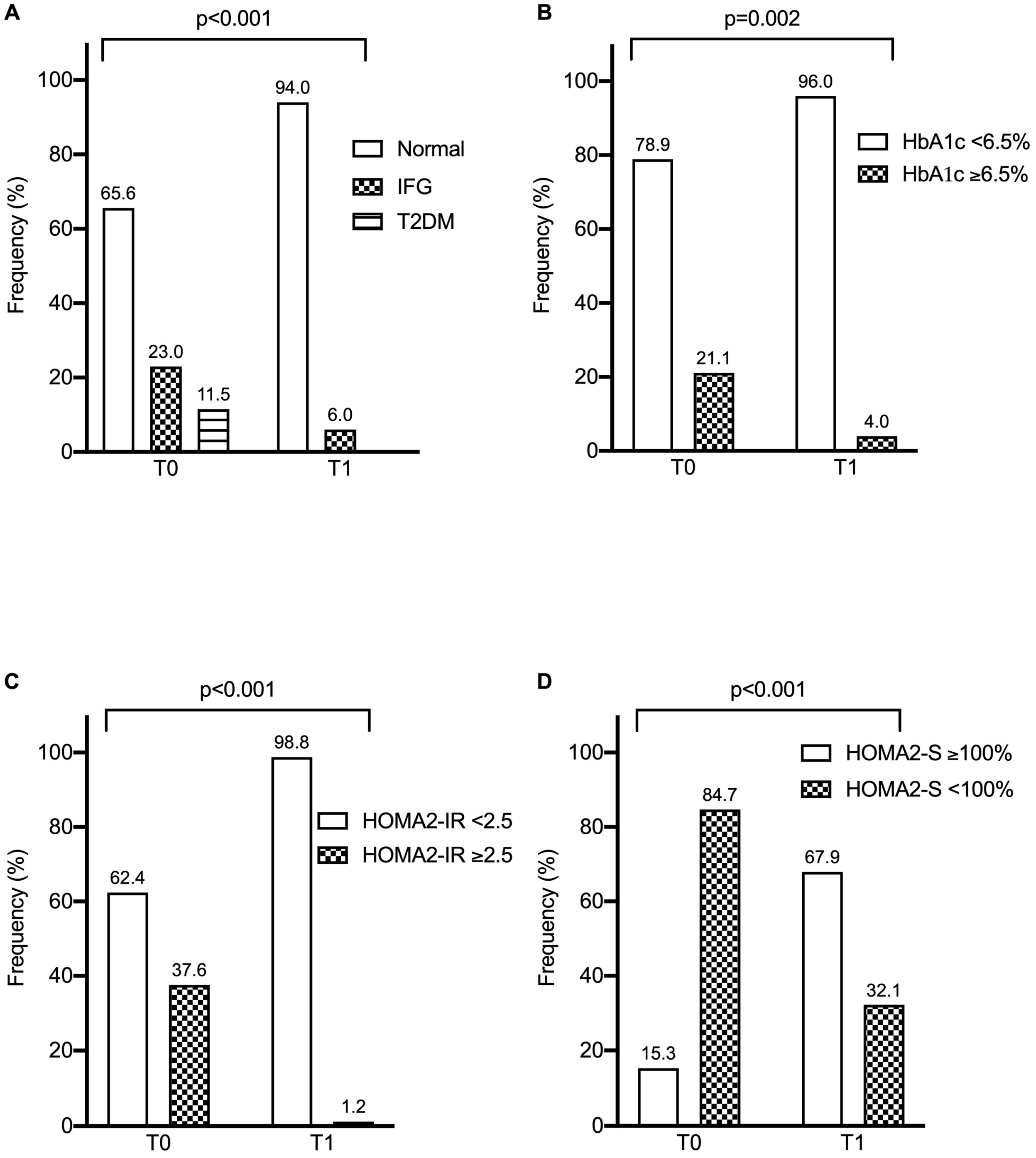

Figure S1. Sleeve gastrectomy significantly improves glycemic profile. Sleeve gastrectomy determine a general improvement of glycemic profile with regression of type 2 diabetes mellitus (T2DM) e reduction of patients with impaired fasting glucose (IFG) (**A**). Accordingly, there was an improvement of serum levels of glycated hemoglobin (**B**), and homeostatic model assessment (HOMA) for insulin resistance (IR) (**C**) and peripheral insulin sensitivity (%S) (**D**).
